# Consistent analysis of differentially expressed genes across 7 cell types in papillary thyroid carcinoma

**DOI:** 10.1016/j.csbj.2023.10.045

**Published:** 2023-10-27

**Authors:** Xianhui Ruan, Yue Huang, Lin Geng, Mengran Tian, Yu Liu, Mei Tao, Xiangqian Zheng, Peng Li, Min Zhao

**Affiliations:** a Department of Thyroid and Neck Tumor, Tianjin Medical University Cancer Institute and Hospital, National Clinical Research Center for Cancer, Key Laboratory of Cancer Prevention and Therapy, Tianjin’s Clinical Research Center for Cancer, Tianjin 300060, China; b School of Science, Technology and Engineering, University of the Sunshine Coast, Maroochydore DC, Queensland 4558, Australia; c State Key Laboratory of Medicinal Chemical Biology, College of Life Sciences, Nankai University, 300071 Tianjin, China; d School of Medicine, Nankai University, Tianjin, China; e Department of Thyroid and Breast Surgery, Tianjin Key Laboratory of General Surgery in Construction, Tianjin Union Medical Center, Tianjin, China

**Keywords:** Papillary thyroid carcinoma, ScRNA-seq, KRT7, EMT, NF-κB

## Abstract

Single-cell transcriptome sequencing (scRNA-seq) provides a higher resolution of cellular differences than bulk RNA-seq, enabling the dissection of cell-type-specific responses to perturbations in papillary thyroid carcinoma (PTC). However, cellular genomic features are highly heterogeneous and have a large number of genes without any expression signals, which hinders the statistical power to identify differentially expressed genes and may generate many false-positive results. To overcome this challenge, we conducted an integrative analysis on two PTC scRNA-seq datasets and cross-validated consistent differential expression. By combining results from 32 common cell types in the two studies, we identified 31 consistently differentially expressed genes (DEGs) across seven cell types, including B cells, endothelial cells, epithelial cells, monocytes, NK cells, smooth muscle cells, and T cells. Functional enrichment analysis revealed that these genes are important for the adaptive immune response and autoimmune thyroid diseases. The additional disease-free survival analysis also confirmed that these 31 genes significantly affected patient survival time in large scale thyroid cancer cohort. Furthermore, we experimentally validated one of the top consistent DEGs as a potential biomarker gene of PTC epithelial cells, *KRT7*, which may be a upstream gene for the NF-κB signaling pathway. The result shows that *KRT7* may promote thyroid cancer metastasis through the epithelial-mesenchymal transition and NF-κB signaling pathway. In summary, our single-cell transcriptome integration-based approach may provide insights into the important role of NF-κB in the underlying biology of the PTC.

## Highlights

• A computational pipeline was constructed to identify the consistent DEGs in PTC single-cell transcriptomes.
• Thirty-one consistently DEGs associated with the adaptive immune response across multiple PTC cell types.
• Identifying KRT7 as a potential biomarker gene of PTC epithelial cells.
• KRT7 promotes thyroid cancer metastasis through the epithelial-mesenchymal transition and NF-κB signaling pathway.

## Graphical Abstract

**Figure:**
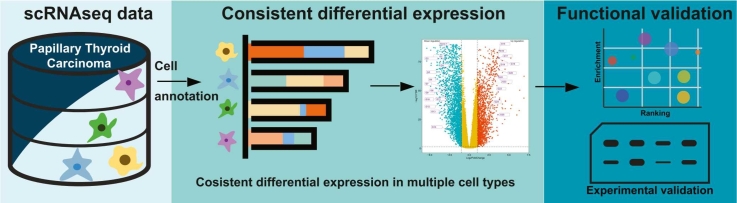

## 1 Introduction

Thyroid cancer is a type of cancer that develops in the thyroid gland, which is located in the neck and is responsible for producing hormones that regulate the body's metabolism. Thyroid cancer is relatively rare compared to other types of cancer, but it is the most common endocrine cancer. There are four main types of thyroid cancer. As the most common type of thyroid cancer, papillary thyroid carcinoma (PTC) accounts for approximately 80% of cases [1]. It typically develops in the cells that produce thyroid hormone and usually grows slowly. The other three types are follicular thyroid cancer (∼10% of cases), medullary thyroid cancer (∼4% of cases), and anaplastic thyroid cancer (<2% of cases) [1]. Although PTC is often curable with surgery and radioactive iodine therapy, the high heterogeneity of clinical and pathological features makes it difficult to develop effective treatments that are applicable to all patients.

Single-cell transcriptome analysis has emerged as a powerful tool for studying the biology of cancer cells at the individual cell level, providing insights into the heterogeneity and complexity of tumor cells [2]. In recent years, several studies have utilized single-cell transcriptome analysis to investigate thyroid cancer. PTC can manifest as multiple spatially distinct tumors composed of functionally distinct nonimmune cell subpopulations, as well as diverse immune cell clusters that interact to influence clinical outcomes. Single-cell RNA sequencing (scRNA-seq) was used to dissect PTC tumor ecosystems related to cancer initiation and progression [3]. A study published in Frontiers in Immunology also revealed that 42 oncogenic signaling pathways and a 6-gene panel predicted the prognosis of PTC and were associated with the tumor immune microenvironment [4].

Identifying differentially expressed genes (DEGs) is one of the typical analysis goals for bulk transcriptome sequencing. Theoretically, DEGs are derived from the comparison of the gene expression in two distinct sample groups. However, DEG analysis at the single-cell level is different from that of bulk sequencing data [5]. The single cell transcriptome may have different individual samples. The research goal is normally to focus on the differential expression in various cell types instead in different individual samples. Therefore, consistent DEGs at the single-cell level indicate the concordant gene expression changes in multiple cell types. In addition, there are more than two groups of cells represented in real scRNA-seq data. Thus, the DEG analysis is more likely to characterize the gene expression difference in a specific cell type compared to the other differences in the remaining cells in the dataset [6]. Due to these differences, identifying consistent DEGs in single-cell transcriptomes will provide novel mechanisms and reliable biomarkers at the cell type level. The results will be more suitable for personalized cell-based or gene-based treatment strategies.

While significant progress has been made in unraveling the molecular mechanisms underlying PTC, there is still much to be learned about the complex interactions between different signaling pathways and how these interactions contribute to cancer development and progression. Advances in this molecular knowledge will also provide more reliable biomarkers for PTC that can accurately predict disease progression or response to treatment. To achieve this goal, we conducted an integrative analysis on two scRNA-seq datasets to explore consistent cell type markers and potential signaling crosstalk in PTC. The validation of the top candidate genes further elucidated the role of KRT7 as a marker in PTC epithelial cells. Overall, we identified consistent marker genes across 30 common cell types in PTC. Moreover, the top 31 genes associated with 7 cell types have a potential clinical application in PTC diagnosis as a source of biomarker discovery. This finding might make it less difficult to develop personalized gene and cellular-based treatment strategies using scRNA-seq technology.

## 2 Materials and methods

### 2.1 Data preprocessing and normalization of scRNA-seq datasets

GEO (Gene Expression Omnibus) is a public functional genomics data repository that supports MIAME-compliant data submissions. To download two PTC-related scRNA-seq datasets from GEO, we collected the accession numbers (GSE184362 and GSE191288) from the two publications [3], [4]. Since we would perform the the data preprocessing, we downloaded the raw matrix files in 10X Genomics cellranger format, which includes three files (barcodes.tsv, genes.tsv and matrix.mtx) for each dataset.

Based on the matrix files, we conducted data quality control-related preprocessing. Quality control is an important step in analyzing scRNA-seq data as it helps to exclude cell barcodes that may not represent intact individual cells and identify potential sources of errors in the experiment [5]. The purpose of data quality control in bulk sequencing is to remove low-quality reads. However, the purpose of our quality control in this study was the removal of low-quality cells, for instance, cells with potential doublets. Then, we ran the data normalization and scale based on a few of the features, for instance, gene expression was normally adjusted according to the cell cycle states.

One common issue that needs to be addressed in scRNA-seq data quality control is the identification and exclusion of doublets, which are generated from two cells and can arise due to errors in cell sorting or capture, particularly in droplet-based protocols involving thousands of cells. Specifically, we utilized Single-Cell Remover of Doublets (Scrublet), a computational framework to identify problematic doublets. In summary, Scrublet eliminates the requirement for expert knowledge or cell clustering by simulating doublets from the data and constructing a nearest neighbor classifier. In addition, we also calculated various metrics such as the number of UMIs, the number of expressed genes, and the proportion of reads mapped to mitochondrial genes using Seurat R package [6].

For the two datasets, we also performed independent data normalization to account for differences in sequencing depth and other sources of technical variation by using the global-scaling normalization method “LogNormalize” developed by Seurat [6]. Using the cell-cycle scoring function in Seurat, we also corrected cell-cycle effects on the transcriptomes [6]. The final regression results were utilized for the subsequent analysis.

### 2.2 Cell clustering and annotation

The scaled cell expression was further clustered by using the CalculateCluster function implemented in the Seurat package [6]. The function uses the graph-based clustering approach implemented by the FindNeighbors and FindClusters functions to set the clustering granularity, with increasing values resulting in a greater number of clusters. Cell clusters are further visualized as t-SNE embeddings on dimension-reduced planes.

Cell type annotation is crucial for the analysis of scRNA-seq data, as it allows us to identify different cell types and understand their functions. Here, we used the Human Primary Cell Atlas (HPCA) database as a reference to annotate our scRNA-seq data. In summary, HPCA is a collection of scRNA-seq datasets for various human primary cells. To annotate human cell types using the HPCA database for scRNA-seq data, we used the SingleR package [7], which annotates scRNA-seq data based on known cell type marker genes. It assigns cell type labels to individual cells based on reference samples with the highest Spearman rank correlations, focusing on the relevant differences between cell types by utilizing only the marker genes between pairs of labels.

Sub-Cluster Identification through Semi-Supervised Optimization of Rare-Cell Silhouettes (SCISSORS) is a powerful tool in scRNA-seq analysis [8]. SCISSORS is intended to identify and characterise rare cell subpopulations within diverse samples. Combining both labelled and unlabeled cells, the method employs semi-supervised learning to maximise the precision of subcluster identification. It begins by generating silhouette scores for each cell, which quantify how well each cell fits within its assigned cluster. In our analysis, we examined this strategy in an effort to gain a deeper comprehension of complex biological systems by revealing hidden cell subpopulations. However, mapping the resulting sub-clusters in two scRNA-seq datasets is difficult.

### 2.3 Consistent differential expression analysis of the single cell transcriptomes

To further refine our data, we extracted the genes with differential expression in PTC datasets independently. In our analysis, we found the top 100 differentially expressed genes via the Seurat function FindAllMarkers [6]. In brief, our selection criteria for the top differentially expressed genes were based on adjusted p values and fold changes. The general overview of the process is as follows: 1) Seurat FindMarkers was used to perform differential expression analysis between groups of cells. By default, Seurat performs differential expression according to the nonparametric Wilcoxon rank sum test between two groups of cells. Seurat also identifies the up- and downregulated genes of a single cell cluster in comparison to all other cells. 2) Genes that are differentially expressed according to the Wilcoxon rank-sum test were identified. 3) To account for multiple testing, p values are adjusted using the Benjamin-Hochberg procedure (FDR correction). Genes with adjusted p values below a specified threshold 0.05 are considered significant. 4) In addition to adjusted p values, genes with a fold change above 1.5 are considered differentially expressed. 5) Finally, the top differentially expressed genes were selected according to their adjusted p values and fold changes. It is important to note that one of the datasets (GSE191288) only had 80 DEGs in our study.

In order to make use of the large number of differential expression results in the two PTC datasets, we combined the heterogeneous datasets to identify genes with common differential expression patterns across various cell types. Thus, consistent positive and negative marker genes for a particular cell type could be identified. Additionally, the combined results provide cross-validation for each other.

### 2.4 Functional enrichment and network analysis

Understanding the biological processes and pathways that are enriched in the consistently differentially expressed genes that we identified, requires functional enrichment analysis. Metascape, a user-friendly and potent web-based tool for functional enrichment analysis, was utilized in this study [9]. The enrichment bar graph from Metascape displays the clustered enrichment functional categories, while the enrichment network visualization shows the intra-cluster and inter-cluster similarities of enriched terms, up to ten terms per cluster. Cluster annotations are shown in a color code. The enrichment heatmap shows the top 20 clusters colored according to the p value, with a darker color indicating a lower p value. In addition, we utilized Metascape to conduct the upstream transcription factor enrichment analysis.

In addition, we also used QIAGEN IPA (Ingenuity Pathway Analysis) [10] to explore the novel pathways not defined in the gene ontology and KEGG databases. After uploading the consistent DEGs to IPA, we performed IPA core analysis to identify the most relevant pathways and functions in our dataset. This will provide us with a summary of the connected top pathways and biological functions that are enriched in our data set. The graphical summary from IPA also provides an overall visualization option by illustrating a combination of canonical pathways, key protein families, up-stream regulators and down-stream target molecules.

By using FunCoup (version 5) [11], human gene-gene interactome was compiled based on large-scale gene co-expression, co-localization, protein-protein interaction, complex co-membership, co-membership in a metabolic pathway, and co-membership in a signaling pathway [12]. The consistent DEGs were mapped to the human interactome and as many connections as possible were made between them. Cytoscape was used to visualize the final output network by incorporating cell type specificity [13].

### 2.5 Survival and clinical analysis using cBio portal

Although single-cell transcriptome analysis has provided valuable insights into the cellular heterogeneities of PTC, the clinical features are still lacking in the cells. Consequently, we analyzed the clinical relevance of the consistently differentially expressed genes in two scRNA-seq datasets by utilizing publicly available large-scale cancer genomics data. In practice, cBioPortal provides a user-friendly interface for exploring genomic and clinical data related to thyroid cancer [14]. It also offers several tools for survival and clinical feature analysis, including the mutational burden, aneuploidy score and prognostic disease free analysis. In this study, we focused on the thyroid carcinoma genomics data from the The Cancer Genome Atlas (TCGA) PanCancer Atlas, which contains 482 samples with single nucleotide and copy number mutations.

### 2.6 Cell culture

The thyroid cancer cell line BCPAP was maintained in RPMI1640 (Gibco) supplemented with 10% FBS and penicillin (100 U/ml)/streptomycin (100 mg/ml) cultured in 37 °C, 5% CO_2_ incubator.

### 2.7 Transfection

Small interfering RNAs (siRNAs) targeting KRT7 and negative controls were synthesized by Sangon Biotech. The siRNA sequences used were as follows: siKRT7–1: 5′- GAAUAAGUACGAAGAUGAAAU-3′, siKRT7–2: 5′- CCCGGAAUGAGAUUUCAGAGA-3′. Transfection was performed according to the manufacturer’s instructions for Lipofectamine 2000 (Gibco). In summary, 20 pmol siRNA was diluted in Opti-MEM (Gibco) and mixed gently. Then 1 μl of Lipofectamine 2000 was diluted in 50 μl of Opti-MEM and incubated at room temperature for 5 min. The diluted siRNA and Lipofectamine 2000 were gently mixed and incubated at room temperature for 20 min. Finally, the mixture was added into the cells.

### 2.8 The transwell cell migration assay

For the migration assay in vitro, BCPAP cells transfected with siKRT7 or NC were plated into transwell inserts precoated with or without Matrigel (BD). The bottom chambers were filled with 200 μl of medium supplemented with 10% FBS. The migratory cells were fixed with 4% fixative solution (Solarbio) and stained with 0.1% crystal violet after incubation at 37 °C for 24 h. Cells were photographed and counted using an inverted microscope in three randomly selected fields.

### 2.9 Western blot

Cells were lysed in RIPA buffer (Beyotime), supplemented with proteinase inhibitor and PMSF, and sonicated. Protein concentration was measured by a BCA protein concentration measurement kit (Beyotime). After adding 5 ×SDS loading buffer, cell lysates were denatured at 95 °C for 5 min, separated by SDS-PAGE, and transferred to PVDF membranes (Millipore). The membrane was blocked in TBST (Tris-buffered saline, 0.1% Tween 20) containing 5% skim milk for 2 h and incubated with primary antibodies (N-CADHERIN, Cell Signaling Technology, 13116 S; E-CADHERIN, Boster Bio, PB9561; VIMENTIN, Cell Signaling Technology, D21H3; P65, Cell Signaling Technology, D14E12; beta-Tubulin, BPI, abm59005–37B-PU) at 4 °C overnight. After three washes, the membrane was incubated with HRP-conjugated secondary antibodies (Mouse IgG HRP Linked Whole Ab, Sigma, NA931V; Rabbit IgG HRP Linked Whole Ab, Sigma, NA934V) at room temperature for 1 h. HRP activity was detected by HRP substrate peroxide solution (Millipore). Digital images were taken with Tanon 5500.

### 2.10 Quantitative real-time PCR

Total RNA was extracted from cells using TRIzol reagent (Invitrogen) according to the manufacturer’s instructions. PrimeScript™ RT Master Mix was used to transcribe RNA to cDNA (Takara). Quantitative real-time PCR (qPCR) was carried out using SYBR Green Mix (Yeasen). Actin served as a control, and the 2^-ΔΔCt^ method was used to evaluate the expression level of each gene. The primers were designed as follows:

SNAIL-Forward: 5′-CGAACTGGACACACATACAGTG-3′, SNAIL-Reverse: 5′- CTGAGGATCTCTGGTTGTGGT-3′, N-CADHERIN-Forward: 5′-TTTGATGGAGGTCTCCTAACACC-3′, N- CADHERIN - Reverse: 5′- ACGTTTAACACGTTGGAAATGTG − 3′, VIMENTIN- Forward: 5′- GAGAACTTTGCCGTTGAAGC − 3′, VIMENTIN- Reverse: 5′- GCTTCCTGTAGGTGGCAATC-3′, P65- Forward: 5′-CCCACGAGCTTGTAGGAAAGG-3′, P65- Reverse: 5′- GGATTCCCAGGTTCTGGAAAC-3′, KRT7- Forward: 5′- CAGGATATGGCACGGCAG − 3′, KRT7- Reverse: 5′- CACAGAGATATTCACGGCTCC-3′.

### 2.11 Immunofluorescence

For cultured cells, cells at approximately 60% confluency were fixed in 4% paraformaldehyde for 30 min, and permeabilized with 0.2% Triton X-100 for 30 min. After blocking in 5% goat serum in PBS for 2 h, cells were incubated with VIMENTIN antibody (VIMENTIN, Cell Signaling Technology, D21H3) overnight at 4 °C and incubated with Alexa Fluor 594 anti-rabbit (Molecular Probe) for 1 h or incubated with Phalloidin-Cy5 Conjugate (APE BIO, B8598) for 30 min at room temperature and then washed with PBS 3 times. Nuclei were stained with Hoechst 33342 (Sigma). Images were captured with a Zeiss Axio-Image Z1 fluorescence microscope.

### 2.12 Statistical analysis

All experimental data were obtained from at least three independent biological experiments and expressed as the mean ± standard error. Experiments were statistically analyzed using an unpaired two-tailed Student’s *t*-test. *P* < 0.05 indicated that the difference was statistically significant. All experimental data were statistically evaluated using GraphPad Prism 7.0.

## 3 Results

### 3.1 Computational workflow to identify consistently differential expression using scRNA-seq data

**Fig. 1:**
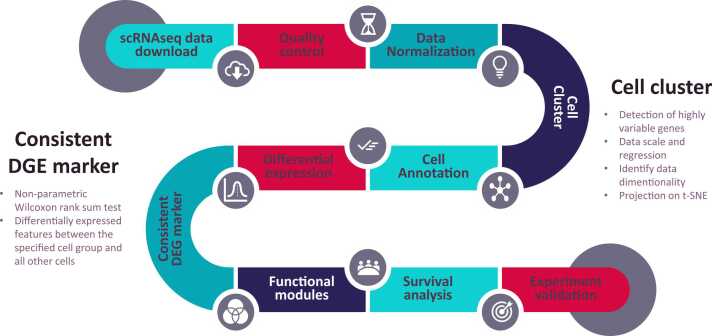
**The workflow to identify and validate the consistently differentially expressed genes in thyroid cancer.** The comprehensive literature review was conducted and filtered out two scRNA-seq datasets related to PTC. The raw matrix files were downloaded to run the quality control and data normalization. By further data scale and regression, we detected the highly variable genes and define the cell clusters. The cell annotation was performed by against human primary cell atlas database. The gene differential expression analysis was conducted by using Seurat for the two datasets. The non-parametric Wilcoxon rank sum tests on the two datasets were conducted to identify those consistent differential expression. A total of 31 consistent DEG markers were identified across 7 cell types. By mapping the 31 key marker genes to the functional and clinical features, we explored the potential prognostic application in thyroid cancer based on survival data. An epithelial marker gene was validated by experimental evidence in thyroid cancer cell lines.

To explore the consistent DEGs in the single cell transcriptomes, we needed to run the scRNA-seq analysis with all the cell types annotated. As shown in the computational workflow (Fig. 1), we started with data quality control. After strict data quality control and data normalization, we highlighted the differential expression on the cell clusters and types. In this study, we focused on PTC, for which two independent scRNA-seq datasets are publicly available. By annotating the cells in the same computational approach, we intend to determine which genes are differentially expressed in specific cell types in two PTC datasets. It is worth noting that our computational pipeline can also be used for multiple scRNA-seq data mining with shared cell types or research goals. The results imply novel and cross-validated cell type specific gene expression.

Finally, our analysis will generate a list of candidate DEGs specific for various cell types. Final experimental validation might be conducted to confirm the hypothetical cell type markers through experiments or other types of OMICs datasets. In our study, we conducted functional and network analyses on the identified consistent DEGs to reconstruct the interplay between different cell types at the single cell level. By taking advantage of large-scale cancer bulk sequencing genomics data, we also explored the potential prognostic features for those cell specific DEGs at the population level. In the end, we prioritized a total of 31 DEGs that were consistent across both datasets. Further experimental validation was performed to demonstrate the significance of a single DEG KRT7 in thyroid cancer cell lines.

### 3.2 Cell clustering and cell annotations in PTC scRNA-seq datasets

In our study, we concentrated on the PTC primary tumor sample. The GSE184362 dataset contains 23 fresh samples from 11 patients, while the GSE191288 dataset contains 3 pairs of bilateral PTC samples and 1 non-tumor sample. To maintain consistency, we utilized only the seven primary PTC samples from GSE184362 and the six PTC samples (three pairs) from GSE191288. The sample with the highest number of cells, 17,703, is T10, while the sample with the lowest number of cells, 4230, is T5. From the seven samples, there were 59,146 cells in total. The six samples of GSE191288 with the highest and lowest number of cells are T3R (5543 cells, sample 3 right side) and T1R, respectively (2447 cells, sample 1 right side). The dataset contains 20311 cells in total.

After completing the cell annotation procedures for each dataset, we annotated 59,146 and 20,311 cells for GSE184362 and GSE191288, respectively (Table S1). By executing the FindClusters function of the Seurat package, we clustered cells on a graph. Notably, resolution is a crucial factor that determines the "granularity" of downstream clustering and must be optimized for each individual experiment. As depicted in Figs. S1–S2, we evaluated three resolutions (0.4, 0.6, and 0.8) that typically result in effective clustering. The mapping relationships between various resolutions revealed that higher resolution values resulted in a larger number of clusters. Using a resolution of 0.8, the GSE184362 dataset contained 29 clusters, and the GSE191288 dataset contained 27 clusters.

To further investigate the distinct molecular mechanisms for each cluster, we identified all potential marker genes for each cluster in two additional datasets (Figs. S3–S4). The Seurat package's FindAllMarkers function was used to identify potential marker genes by comparing each cluster to all other clusters. Each cluster's cells were treated as replicates, and a differential expression analysis was conducted. It is important to note that these marker genes were cluster-specific and differed slightly from the marker genes identified after cell annotation. Some clusters were merged because they were derived from the same cell types (Fig. S5–S6).

**Fig. 2:**
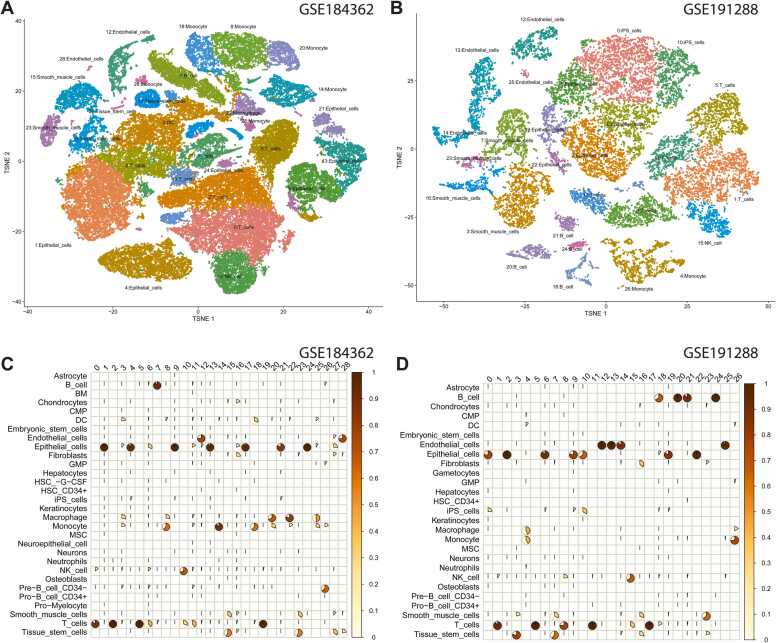
**Cell clustering and annotation for two PTC scRNA-seq datasets.** The t-SNE visualization of cells colored by cluster membership for (**A**) GSE184362 and (**B**) GSE191288. The cluster ID were marked as numbers in the corresponding t-SNE map. The pie-chart plots depict the proportion of cell types for each cell cluster in (**C**) GSE184362 and (**D**) GSE191288.

**Fig. 3:**
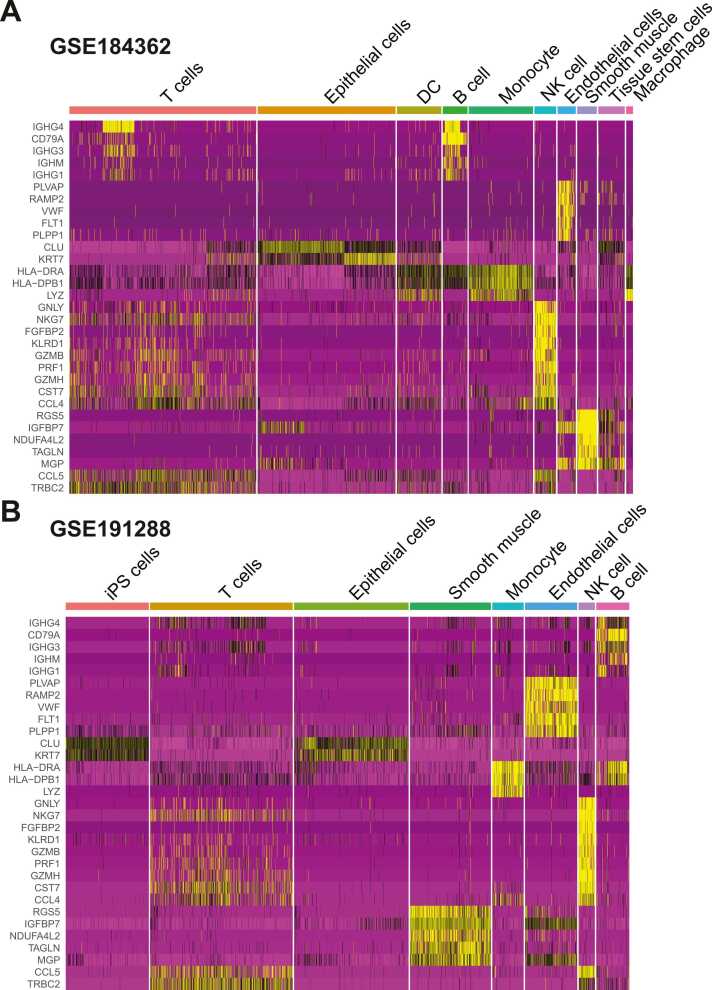
**The expression heatmap of 31 genes consistently differentially expressed in two PTC scRNA-seq datasets: GSE184362 (A) and GSE191288 (B).** The cell types exhibited distinct coloring.

In general, based on reduced data dimensions (t-SNE), the clustering patterns of cells were similar in two independent datasets (Fig. 2A-B). In total, we annotated 30 cell types in GSE184362 and 27 cell types in GSE191288. In total, there were 31 unique cell types and 26 common cell types (Table S1). According to the cell type-specific marker genes, cells from the same cell clusters could be annotated as distinct cell types (Fig. 2C-D). For instance, the majority of the cells in Cluster 1 (GSE184362) were T cells, but a small number of NK cells were also detected in Cluster 1. Therefore, we could easily identify the predominant cell types for each cluster of cells from Fig. 2C-D (Table S1). For clusters with a small number of cells, the predominant cell type was selected by analyzing all marker genes for each cluster in the two datasets (Fig. 3A-B). In practice, the cell type of a cluster is represented by the most significant marker genes that are found in the majority of cells in the cluster. Consequently, our analysis focuses primarily on clusters with well-annotated cell types for the vast majority of cells. However, it is important to note that some cell types, such as epithelial and endothelial cells, were found in multiple clusters.

### 3.3 The expression and cell specificity of 31 consistent DEGs across 7 cell types

On the basis of the well-annotated cell types, the DEG analysis was repeated. In this way, the identified DEGs were the potential marker genes for specific cell types instead of cell clusters. In our study, the top 100 DEGs were extracted from both datasets. Notably, the GSE191288 analysis resulted in only 80 DEGs, whereas the GSE184362 analysis resulted in 110 DEGs (Table S2). By mapping both the gene symbols and cell types, a total of 31 consistent DEGs across seven cell types were identified (Table S2). In summary, mapping and integration at the gene level allow independent datasets to cross-validate each other, which is useful for identifying single-cell-based marker genes.

**Fig. 4:**
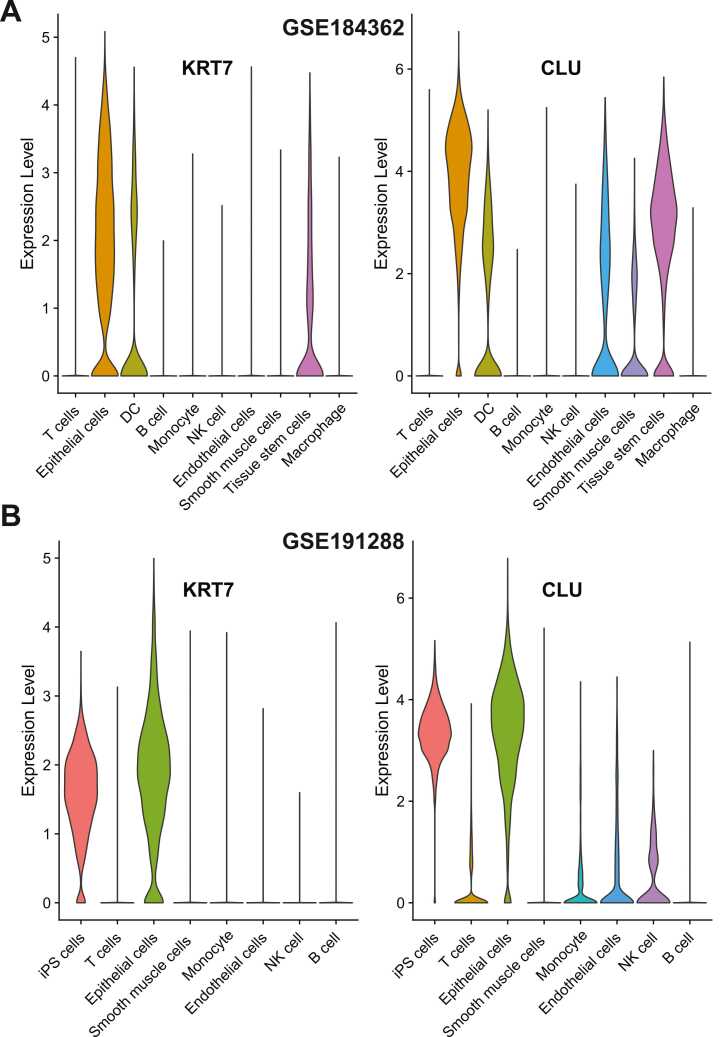
**The violin plots of*****KRT7*****and*****CLU*****present the expression level across multiple cell types for two PTC scRNA-seq datasets.** The expression of the KRT7 and CLU are summarized for (**A**) GSE184362 and (**B**) GSE191288.

As shown in Table S2, the 31 DEGs were specific to seven distinct cell types. Although NK cells were not the most abundant cell type, there were nine DEGs that were consistent in two independent scRNA-seq datasets. Five DEGs were identified in B cells, endothelial cells, and smooth muscle cells. In addition to the three DEGs identified in monocyte, T cells and epithelial cells each contained two DEGs. *KRT7* and *CLU*, two DEGs specific to epithelial cells (Fig. 4A-B), were highly expressed in epithelial cells across both datasets. In addition, we discovered that both genes were expressed in tissue stem cells in the GSE184362 dataset and in iPS cells in the GSE191288 dataset. The iPS cell is similar to the tissue stem cell, which may imply that both genes are important for cell-based cancer development. In summary, the identification of the DEGs based on cell type may provide credible evidence regarding the dependable mechanism during the progression of PTC.

### 3.4 The prognostic features of 31 consistent DEGs across 7 cell types

**Fig. 5:**
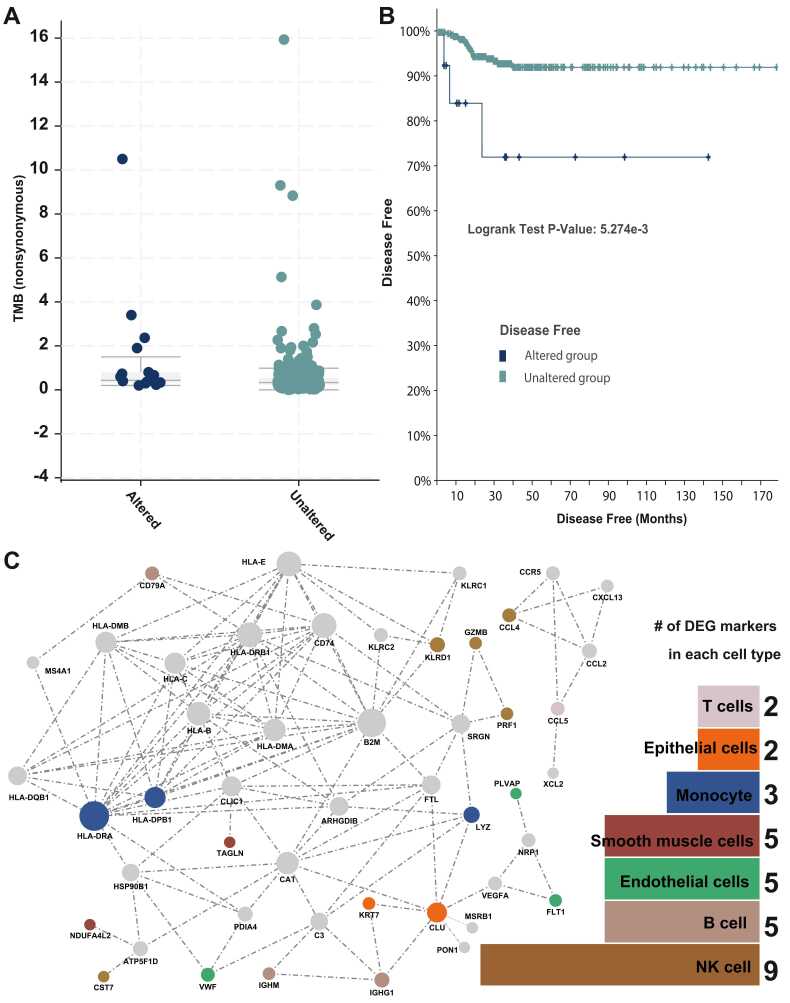
**The clinical and network feature for the 31 consistently differentially expressed genes.** (**A**) Based on the TCGA thyroid cancer dataset, the tumor mutational burden (TMB) patterns for those samples with and without genetic mutations in the 31 genes. (**B**) Analysis of disease-free survival for patients with and without mutations in 31 genes. (**C**) Based on the 31 genes, a functional subnetwork was reconstructed. The distribution of the 31 genes across cell types is depicted in the bar chart on the right. The network node color is identical to the color code used in the bar chart.

To investigate the potential application of PTC prognosis, we mapped the 31 consistent DEGs to the TCGA thyroid genomics data comprising 482 samples in total (Fig. 5A). By examining the clinical characteristics of the thyroid cohorts in greater depth, we were able to divide the thyroid cancer cases into two distinct groups based on the mutational status of the 31 genes. As shown in Fig. 5A, the nonsynonymous tumor mutational burden differed between the two sample groups (Wilcoxon test p value = 5.935E-3). To explore if the more loss-of-function burden, we also run the Wilcoxon test for mutation count and aneuploidy score in the entire TCGA PTC cohort. However, the Wilcoxon test of all the mutation count is approximately 0.021 and the p value of aneuploidy score is 0.150. In Fig. 5B, log-rank test p value = 5.274E-3) demonstrates that additional analyses of survival indicate that the group without genetic mutations can survive for a relatively longer period of time than the group with mutations.

Network biology has facilitated progress in many areas of biomedical science. This simple, yet powerful, concept allows us to abstract the essence of relations between genes and discover important associations. To gain a deeper understanding of the functional links of the 31 genes at the system level, we reconstructed their gene-gene interaction network (Fig. 5C). There are a total of 48 genes in the network, 29 of which are bridge genes that connect the remaining 19 genes from our 31 input genes. Five of the nine DEGs that are specific to NK cells are connected in the reconstructed network. Consequently, B cells, endothelial cells, and monocytes each contain three DEGs. In addition, two DEGs were linked to two cell types (smooth muscle cells and epithelial cells) in the network. Despite the fact that these 31 genes play important roles in various cell types, they have potential prognostic utility as a whole.

### 3.5 The functional features of the 31 consistent DEGs

**Fig. 6:**
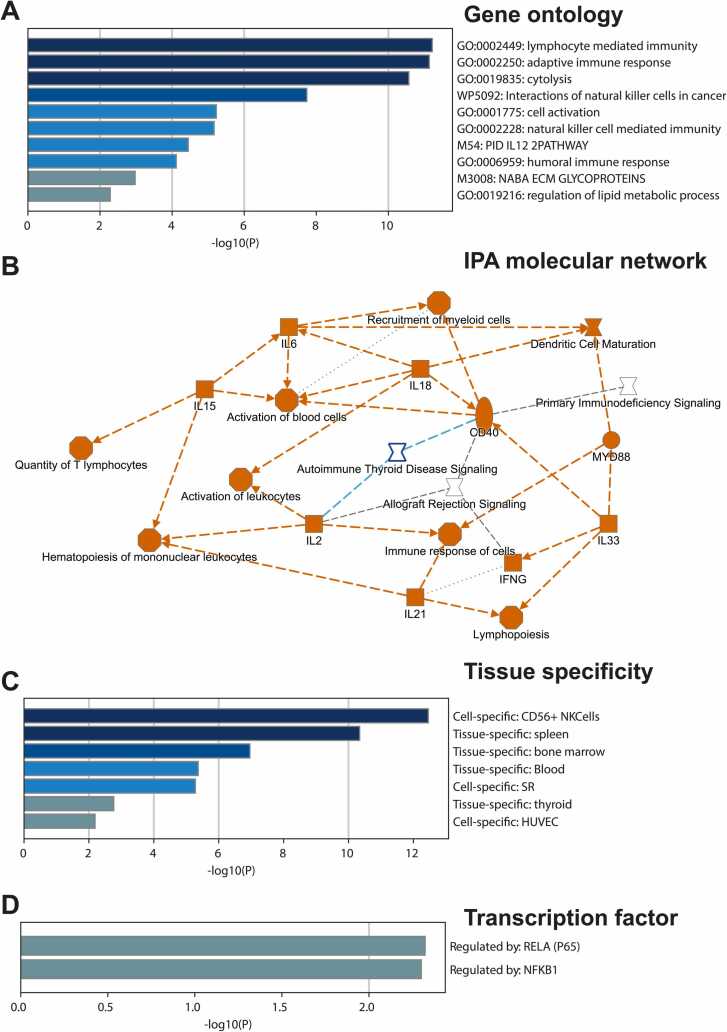
**The functional enrichment analysis for the 31 consistently expressed genes in two PTC datasets.** (**A**) the top over-represented gene ontology terms. (**B**) the IPA network summary for the top over-represented canonical pathways, regulators, and the 31 DEGs. (**C**) the tissue specificity based on the large-scale gene expression data. (**D**) the transcription factor regulation enrichment.

Since these 31 differentially expressed genes are from various cell types, we are interested in determining their shared functions. We conducted the functional enrichment analysis using MetaScape [9] and IPA [10] to accomplish this goal (Fig. 6). As depicted in Fig. 6A, the majority of the ten most overrepresented gene ontology terms and pathways are associated with the immune response, including lymphocyte-mediated immunity, adaptive immune response, and cytolysis. Lymphocyte-mediated immunity is a type of adaptive immune response that involves the activation of T and B cells [15]. Cytolysis is a process by which cells are destroyed by the immune system [16]. In fact, cytolysis is associated with lymphocyte-mediated immunity. In general, lymphocytes of the adaptive immune response must interact with antigen-embedded MHC class II molecules to mature into functional immune cells. Cytotoxic T cells mediate one arm of the cellular immune response. There are two main types of T cells: helper T lymphocytes and cytotoxic T lymphocytes. T H lymphocytes function indirectly to tell other immune cells about potential pathogens, while cytotoxic T cells are the key component of the cell-mediated part of the adaptive immune system that attacks and destroys infected cells [16].

Compared to the gene ontology and common pathway databases such as KEGG, the QIAGEN IPA contains tens of thousands of small pathway terms with specific foci (Table S3). By zooming out using the IPA pathway (Fig. 6B), we discovered that 31 genes are related to the signaling of autoimmune thyroid disease and allograft rejection. In addition, these 31 genes may play vital roles in the activation of blood cells and leukocytes, the recruitment of myeloid cells, and the quantity of T cells. Several cytokines, including IL6, IL15, IL18, IL21, and IL33, may serve as the molecular link between these diverse pathways. Further tissue specificity analysis (Fig. 6C) also revealed the that these consistent DEGs are more likely to be enriched in NK cells, blood cells and the thyroid gland.

We also conducted an enrichment analysis of upstream transcription factors. The results indicate that *RELA* and *NFKB1* are potential regulators for several of the consistent DEGs. *RELA*, also known as p65, is a protein that participates in the formation, activation, and nuclear translocation of the nuclear factor-kappa B (NF-κB) transcription factor complex, which is essential for the regulation of numerous cellular processes, such as cellular metabolism and chemotaxis [1]. *RELA* and *NFKB1* are both involved in immune response regulation. *RELA* is essential for TCR signaling and naive T-cell activation, and aberrant NF-B activation can lead to aberrant T-cell activation.

Although *RELA* and *NFKB1* have not been directly linked to the development of thyroid cancer, the protein has been implicated in the development of other types of cancer. The significance of *NFKB1* as a suppressor of the NF-κB response is evident in mouse models, where Nfb1(-/-) mice exhibit increased inflammation and susceptibility to certain forms of DNA damage, leading to cancer and an accelerated aging phenotype [17]. In gastric carcinogenesis, the basic expression and functional role of *NFKB1* and *RELA* (components of the canonical NF-κB pathway) have been studied, and their regulation by microRNAs has been explored [18]. Based on the known transcription factor binding data, there are three known target genes (*CCL4*, *CCL5* and *VWF*) among the 31 DEGs. More interestingly, another gene *KRT7* is also linked with the NF-κB pathway via microRNA − 199a [19]. In summary, while *RELA* and *NFKB1* have been shown to play a role in the development of various types of cancer, their specific function in thyroid cancer development remains unclear.

### 3.6 The experimental links of *KRT7* to EMT and the NF-κB signaling pathway in thyroid cancer cells

We conducted a literature review and biomarker annotation to further investigate the potential molecular mechanisms and to locate useful biomarkers for PTC (Table S4). We found six genes that could be used as diagnostic, prognostic, or efficacy biomarkers related to PTC among the 31 DEGs that were consistent across all samples. Intriguingly, all six of these associations with PTC are based primarily on computational results from a meta-analysis of bulk sequencing transcriptomes [20]. It is encouraging that differential expression analysis based on bulk sequencing yields similar results to our scRNASeq-based results. To further solidate our result, we chose a single gene to validate the remaining DEGs with unknown biomarker application based on an exhaustive literature review and the absence of a direct link to PTC.

Specifically, we focused on the gene, *KRT7* (keratin 7), which is a type of intermediate filament protein that is typically expressed in simple epithelial cells. In our study, the *KRT7* is a DEG derived from the epithelial cells. In other cancer cells, up-regulation of *KRT7* expression has been observed in various types of cancer, including breast, ovarian, lung, and pancreatic cancers [21], [22]. The up-regulation of *KRT7* may be associated with several biological processes related to cancer progression, including increased cell migration, invasion, and metastasis. Moreover, *KRT7* overexpression may contribute to the acquisition of a more mesenchymal phenotype, which is associated with increased invasiveness and resistance to therapy. Therefore, the up-regulation of *KRT7* expression in cancer cells may be a potential diagnostic and therapeutic target for the treatment of various cancers.

**Fig. 7:**
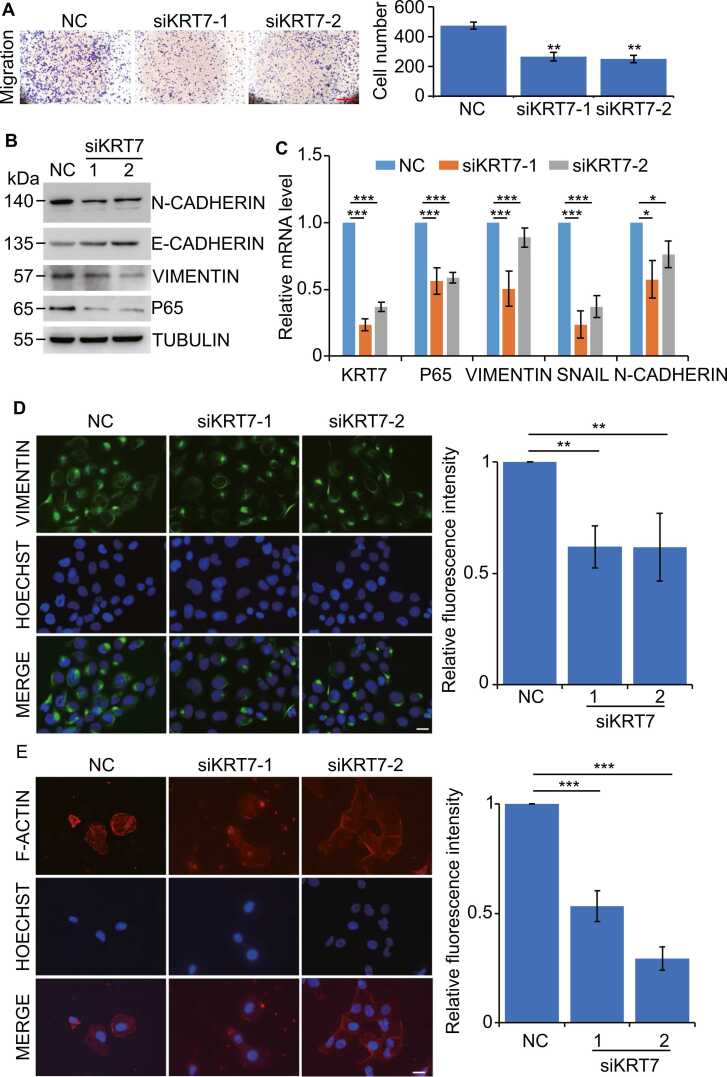
**Knockdown of*****KTR7*****undermines cell motility and reverses EMT process. (A)** Transwell assay was preformed to show that KD *KRT7* attenuated significantly migration abilities of BCPAP. Scale bars, 20 µm. The statistic was analyzed on the right individually. **(B)** siKRT7 was transfected to BCPAP for 48 h and protein levels of N-CADHERIN, E-CADHERIN, VIMENTIN, P65 were analyzed by Western blot. **(C)** siKRT7 was transfected to BCPAP for 48 h and mRNA levels of KRT7, P65, VIMENTIN, SNAIL, N-CADHERIN, were analyzed by RT-qPCR. **(D and E)** The levels of VIMENTIN (D) and F-ACTIN (E) were assessed by immunofluorescence in siKRT7 treated BCPAP cells. Scale bars, 20 µm. Relative fluorescence intensities of VIMENTIN and F-ACTIN were shown on the right. Data in this figure, mean ± SD, *P < 0.05, * *P < 0.01, * **P < 0.001.

In order to gain a comprehensive understanding of the role that *KRT7* plays in the progression of thyroid cancer, we conducted a functional study in thyroid cancer cells (Fig. 7). The results from the transwell assays demonstrated that the migration of thyroid cancer cells was significantly retarded by inhibiting the expression of *KRT7* (Fig. 7A). Knockdown of *KRT7* can block the key genes of epithelial-mesenchymal transition (Cadherin and Vimentin), as well as the key genes of NF-κB signaling pathways (P65), as shown by additional positive western blot results in Fig. 7B. Similar tendencies were also validated by qPCR results (Fig. 7C). For instance, the decrease of *KRT7* led to an inhibition of the NF-κB.

The results of our analysis of the enriched transcription factor in PTC indicated that p65 and NFkB1 were upregulated (Fig. 6D). It has been reported that p65 degradation impairment promotes epithelial-to-mesenchymal transition [23]. Generally, p65 is known to be a member of the NF-B family. In ovarian cancer, *KRT7* overexpression was associated with EMT. It was suggested that the mechanism of *KRT7* regulating EMT was linked with p65. Fig. 7A and Fig. 7B confirm that *KRT7*, p65, and EMT markers such as SNAIL and N-CADHERIN are downregulated. All of the results demonstrated the relationships between *KRT7*, cell migration, and EMT. In Fig. 7D, we used immunofluorescence to provide further evidence that inhibiting the activity of *KRT7* led to a reduction in the amount of Vimentin and cytoskeletal proteins that were produced. When considered as a whole, the results of all of these experiments suggested that inhibiting the activity of *KRT7* resulted in a reduction in the migration of thyroid cancer cells via the EMT and NF-κB signaling pathways.

## 4 Discussion

Our established computation pipeline started from consistent differential expression analyses at the single cell level. It is important to note that our computational approach is one general procedure that can be used to identify functional genes, and the specific steps and methods used will depend on the specific research question being asked and the availability of the scRNA-seq data. In bulk sequencing transcriptomics-based biomarker analysis, the strategies are mostly based on patient groups, such as gene differential expression analysis. The scRNA-seq provides an ideal source to detect some essential genes associated with cancer cell differentiation at the single cell level. Therefore, individual-based scRNA-seq analysis is more practical for personalized cancer management. Our approach here could be an efficient choice for cell type specific biomarker discovery by using a combination of scRNA-seq and individual-based molecular evidence.

The existing advanced algorithms and tools for integrating single-cell data include iDESC and scMerge. scMerge is designed to identify differential gene expression in multiple single-cell datasets [24], whereas iDESC is used to identify differential expression in single-cell RNA sequencing data from multiple subjects [25]. Instead of combining the independent datasets under the assumption that their reference genes are expressed at the same level, our comparison was performed at the gene and cell type levels. In our pipeline, we performed quality control and normalization on each dataset individually to eliminate any technical variations using the Seurat function NormalizeData. After normalization, we mapped the genes and cell types between the two datasets to identify the DEGs shared by specific cell types. Therefore, the DEG analyses in each dataset were normalized and batch effects were not taken into account. Our method eliminates the need to account for the always-present batch effect and unknown technical variations in multiple datasets. Multiple independent datasets were subjected to the same computational pipeline, with integration occurring after the cell annotation stage. Cross-validation in two independent datasets was provided by the integration at the gene level. In summary, by following these steps and utilising Seurat's functions, we effectively integrated multiple single-cell transcriptome datasets at the gene level for more accurate analysis and biological insights in subsequent steps.

In our study, the presence of multiple clusters within a single cell type may indicate biological heterogeneity in the development of PTCs. Gene expression can vary among cells of the same type due to factors such as cell cycle phase, cellular activation state, and subtle environmental variations. Within a seemingly uniform cell type, different clusters may represent distinct subtypes or activation states. This can shed light on the functional diversity of seemingly identical cell populations. The presence of multiple clusters within the same cell type demonstrates the complexity and diversity of biological systems as a whole. To draw meaningful conclusions from scRNA-seq data, researchers must carefully interpret these clusters, taking both biological and technical factors into consideration. To address these obstacles and refine cell type annotations, advanced analytical techniques and integration methods are frequently applied.

Data on the transcriptome of a single cell can reveal a high degree of cellular heterogeneity within a tissue or tumour. This heterogeneity is caused by genetic, epigenetic, and microenvironmental differences, resulting in numerous subpopulations. Typically, cell types exist along a continuum rather than in discrete categories. Gene expression can exhibit gradations, making it challenging to define distinct boundaries between cell types. Depending on their research objectives and available resources, researchers may choose to focus on both the main cell population and subpopulations in many instances. We focused our analysis on the predominant cell population. However, we also investigated the possibility of merging clusters that contain the same major cell types, such as T cells. As depicted in Fig. S7, the six clusters related to T cells (0, 2, 5, 6, 11, and 19 as depicted in Fig. 2) in the GSE184362 dataset could be sub-clustered into nine sub-clusters. The clusters (1, 5, 8, 11, and 17 as shown in Fig. 2) in the GSE191288 dataset consist primarily of T cells that were sub-clustered into 10 groups. Although these new sub-clusters may be useful for the T cells' heterogeneous characteristics, it is difficult to map them to each other in the two independent datasets. For example, the marker gene CCR7 is only expressed in sub-cluster 2 of GSE184362, whereas there are three sub-clusters in GSE191288 (4, 7, and 8). Therefore, it is difficult for these small subclusters to share the DEG marker across multiple independent datasets. These data were presented in this manuscript; however, the result did not align well with our original objective, which was to identify the common DEG marker in the typical PTC cell types. In sum, for identifying and characterising subpopulations in single-cell transcriptome data, advanced computational methods, such as sub-clustering algorithms, are essential. The choice of methodology is ultimately determined by the specific research questions and the biological context of the study.

In our study, we identified two transcription factors from the NF-κB signaling pathway, which is a ubiquitous molecular network and involved in inflammatory and immune responses, as well as in the regulation of the expression of many other genes related to cell survival, proliferation, and differentiation [26]. An emerging body of evidence shows that NF-κB plays a crucial role in thyroid cancer, including cancer development and progression [27]. Increased activity of NF-κB has also been observed in thyroid cancer, where it correlates with a more aggressive pattern [26].

P65 which is a subunit of NF-κB has a transcriptional activation domain and is involved in cell survival, invasion, proliferation, metastasis, angiogenesis, and chemoresistance [28]. Ubiquitination of p65 plays an important role in regulating its function. In ovarian cancer cells, p65 binds to the mortalin promoter and promotes ovarian cancer cell proliferation and migration by regulating mortalin [29]. In thyroid cancer cells, oncogene activation prevented TGF-ß/SMAD-dependent p27 repression and CDK2/SMAD3 phosphorylation, leading to p65 up-regulation, which repressed BAX, induced cyclin D1 and promoted TGF-ß-dependent growth [30].

Keratin 7 (KRT7) is a member of the keratin gene family. *KRT7* is abnormally expressed in various types of cancer and promotes the malignant progression of tumors [21], [22]. In ovarian cancer, *KRT7* overexpression was associated with increased proliferation, migration and EMT of ovarian cancer cells [21]. *KRT7* regulates EMT in ovarian cancer via the TGF-ß/Smad2/3 pathway, and regulates cell-matrix adhesion through integrin-ß1-focal adhesion kinase signaling [21]. However, there is a lack of specific information regarding the role of *KRT7* in thyroid cancer development. In our study, we are the first to link the *KRT7* to EMT and the function of P65, which is the core molecule in the NF-κB signaling pathway.

Based on an exhaustive literature review and the absence of a direct link to PTC, we chose a single gene to validate the marker for the 31 consistent DEGs. However, only 6 of the 31 genes have known biomarker applications. Due to the insufficient and limited experimental efforts required to validate every function and potential mechanism, only *KRT7* was selected for further analysis. We believe that the remaining 24 genes without known biomarker applications will be crucial for cell-based disease analysis and biomarker discovery.

In our network analysis, we also discovered that 31 DEGs were enriched in autoimmunity to the thyroid gland. In fact, autoimmune thyroid disease, particularly Hashimoto's thyroiditis (HT) and PTC have some links. Those patients with Hashimoto's thyroiditis, for instance, have an increased risk of developing PTC [31], [32], [33]. On a molecular level, the inflammatory processes and cytokines generated by autoimmune thyroid diseases may foster the development of thyroid cancer. Interleukin-6 (IL-6) and tumor necrosis factor alpha (TNF-alpha) may play a role in the progression of tumors. In summary, our results confirmed that autoimmune thyroid diseases may be associated with an increased risk of developing PTC due to the interaction of inflammation, genetic factors, and microenvironmental changes. Moreover, our network analysis links allograft rejection to PTC. Recipients of solid organ transplants are reported to have a higher risk of developing PTC than the general population [34], [35]. This elevated risk is attributed to various factors, including long-term immunosuppressive therapy, which is administered to prevent allograft rejection. Immunosuppression can impair the immune system's capacity to detect and eliminate cancerous cells. Together, these details will aid in the early diagnosis and treatment of patients with PTC, organ transplant, and autoimmune thyroid disease.

## 5 Conclusion

In conclusion, our systematic analysis of the transcriptome of a single cell revealed a total of 31 consistently differentially expressed genes associated with the adaptive immune response in thyroid cancers. KRT7 was consistently differentially expressed in PTC tissues and was associated with inferior clinical characteristics of thyroid cancer patients, as demonstrated by the additional experimental validation. Through extensive molecular interactions with EMT and the NF-κB signaling pathway, the downregulation of KRT7 was found to inhibit cancer cell migration, as determined by a mechanism analysis. In addition, these consistently differentially expressed genes provided insight into the development and progression of PTC and may provide a promising biomarker using single cell-based technology for PTC.

## Competing interests

The authors have declared no competing interests.

## Appendix A Supplementary material

**Figure S1**. The detailed clustering processing parameters used by GSE184362. The mapping of cell clusters according to three parameter resolutions (0.4, 0.6, and 0.8) is depicted in distinct colors. The size of the nodes was proportional to the number of cells in each cluster.

.

**Table S3.** The Ingenuity® Pathway Analysis (IPA®) analysis of the 31 consistently differentially expressed genes in two PTC scRNA-seq datasets.

.

**Table S4.** The clinical application and drug information of the 31 consistently differentially expressed genes in two PTC scRNA-seq datasets.

.

**Figure S2**. The detailed clustering processing parameters used by GSE191288. The mapping of cell clusters according to three parameter resolutions (0.4, 0.6, and 0.8) is depicted in distinct colors. The size of the nodes was proportional to the number of cells in each cluster.

.

**Figure S3**. The heatmap patterns for the top 100 DEGs across multiple cell types in the GSE184362 dataset.

.

**Figure S4**. The heatmap patterns for the top 100 DEGs across multiple cell types in the GSE191288 dataset.

.

**Figure S5**. The phylogenetic trees for the similarities between the cell clusters in GSE184362 dataset.

.

**Figure S6**. The phylogenetic trees for the similarities between the cell clusters in GSE191288 dataset.

.

**Figure S7.** Sub-clustering of T cells for two PTC scRNA-seq datasets. The UMAP visualization of T cells colored by Louvain sub-cluster for (**A**) GSE184362 and (**B**) GSE191288. The cluster ID were marked as numbers in the corresponding UMAP map. The violin plots depict the key marker genes for each cell cluster in (**C**) GSE184362 and (**D**) GSE191288.

.

**Table S1.** The cell type annotations for two PTC scRNA-seq datasets.

.

**Table S2.** The 31 genes with consistently differential expression between two PTC scRNA-seq studies.
