# Supplementary figures and images for: Consistent analysis of differentially expressed genes across 7 cell types in papillary thyroid carcinoma
Source: Comput Struct Biotechnol J. 2023 Oct 27;21:5337–49. doi: 10.1016/j.csbj.2023.10.045 (PMC10637855; doi:10.1016/j.csbj.2023.10.045)

GSE184362

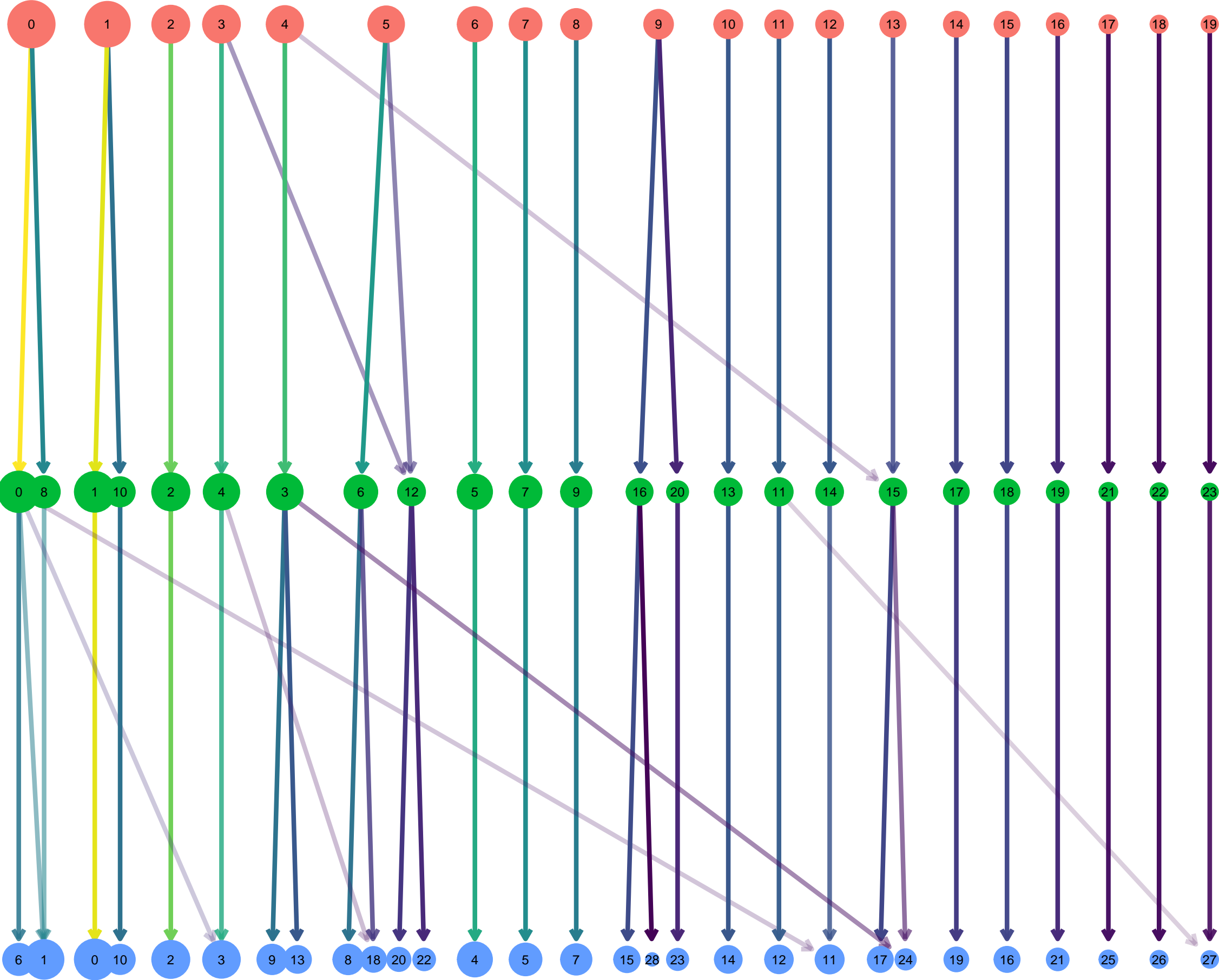

Supplement: Supplementary file 1 — Figure S1. The detailed clustering processing parameters used by GSE184362. The mapping of cell clusters according to three parameter resolutions (0.4, 0.6, and 0.8) is depicted in distinct colors. The size of the nodes was proportional to the number of cells in each cluster. [file mmc1.pdf]

GSE191288

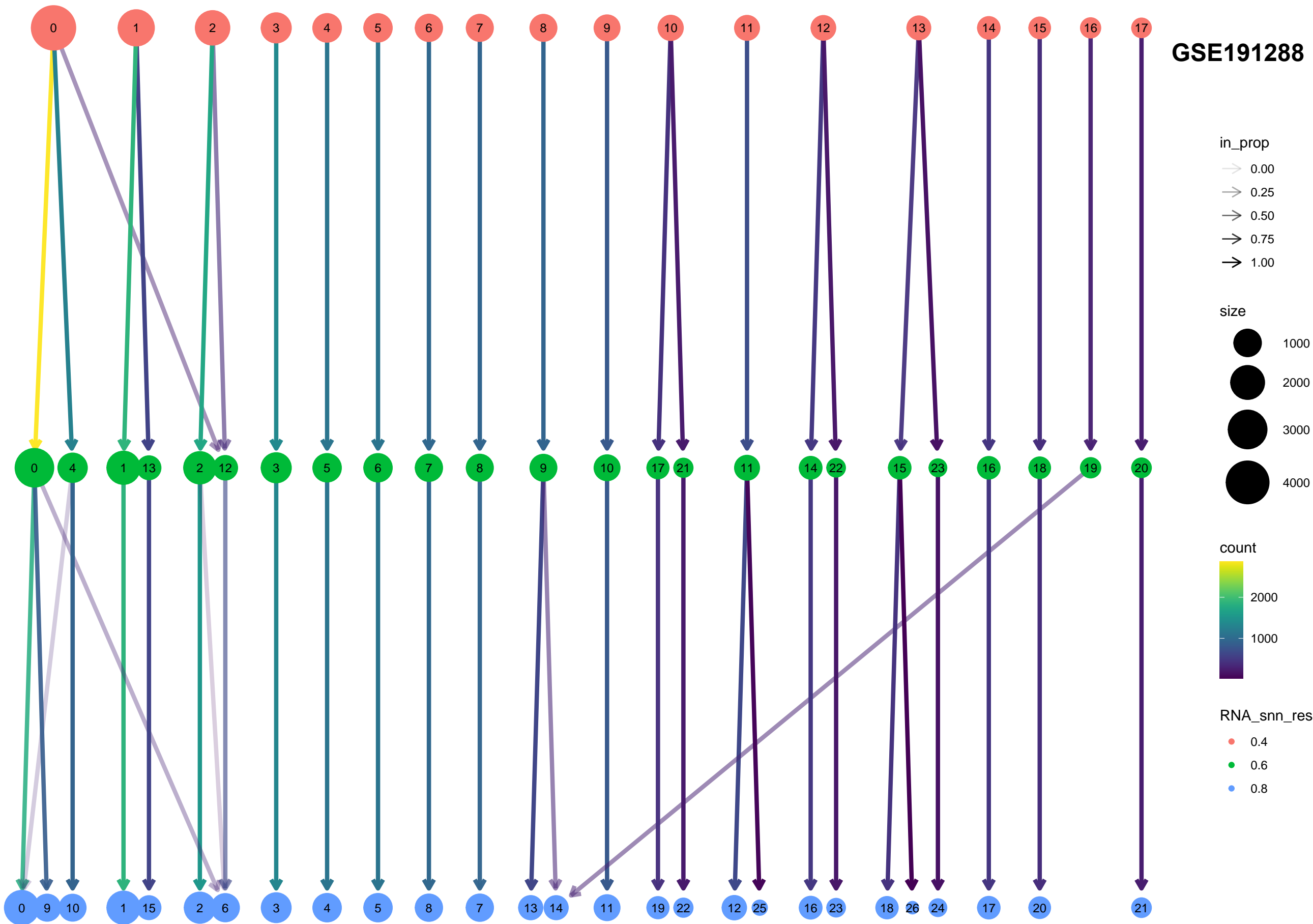

Supplement: Supplementary file 4 — Figure S2. The detailed clustering processing parameters used by GSE191288. The mapping of cell clusters according to three parameter resolutions (0.4, 0.6, and 0.8) is depicted in distinct colors. The size of the nodes was proportional to the number of cells in each cluster. [file mmc2.pdf]

**GSE184362** 0 1 2 3 4 5 6 7 8 9 10 11 12 13 14 15 16 17 18 19 20 21 22 23 24 25 26 27 28 29 30 31 32 33 34 35 36 37 38 39 40 41 42 43 44 45 46 47 48 49 50 51 52 53 54 55 56 57 58 59 60 61 62 63 64 65 66 67 68 69 70 71 72 73 74 75 76 77 78 79 80 81 82 83 84 85 86 87 88 89 90 91 92 93 94 95 96 97 98 99

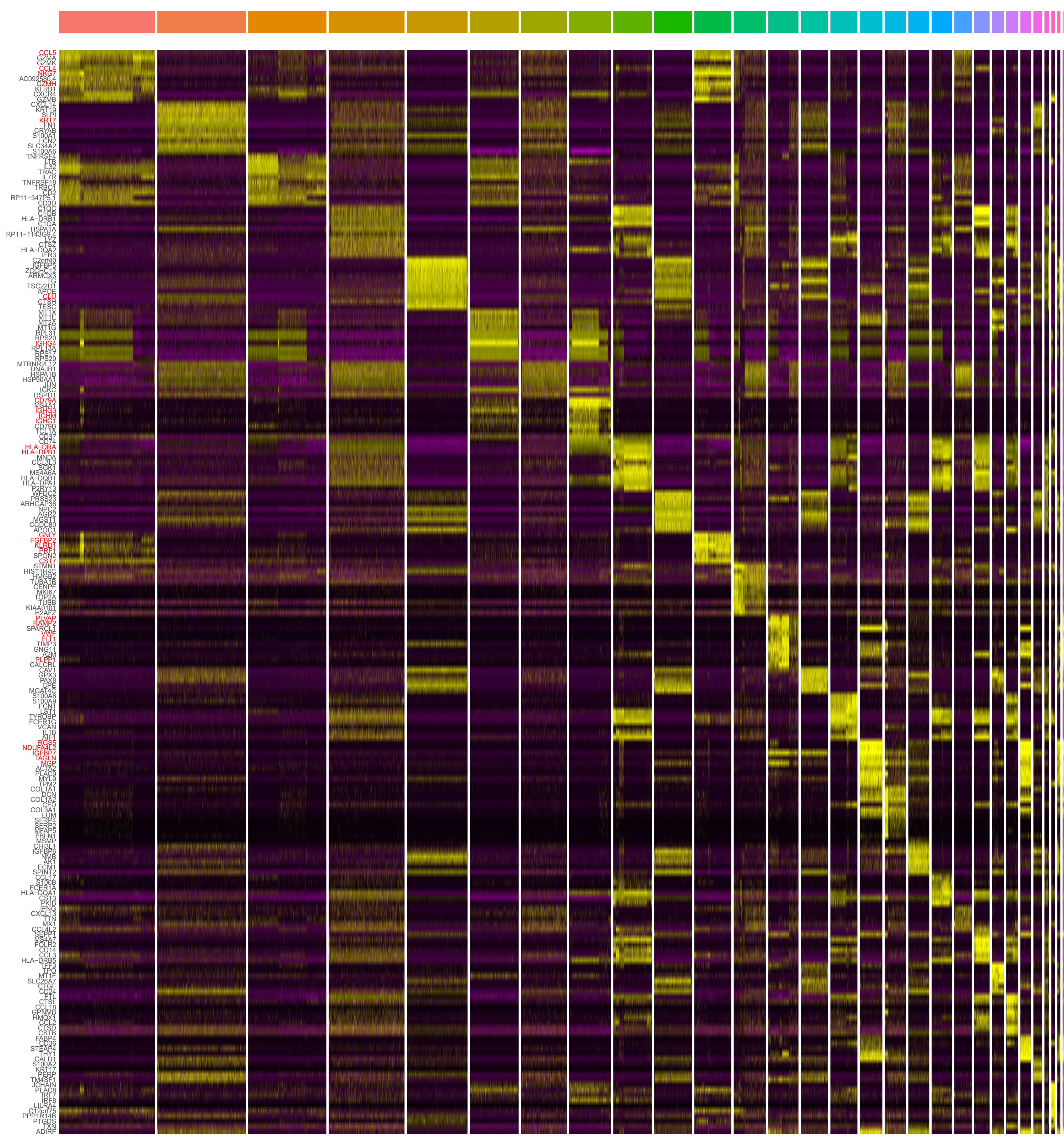

Supplement: Supplementary file 5 — Figure S3. The heatmap patterns for the top 100 DEGs across multiple cell types in the GSE184362 dataset. [file mmc3.pdf]

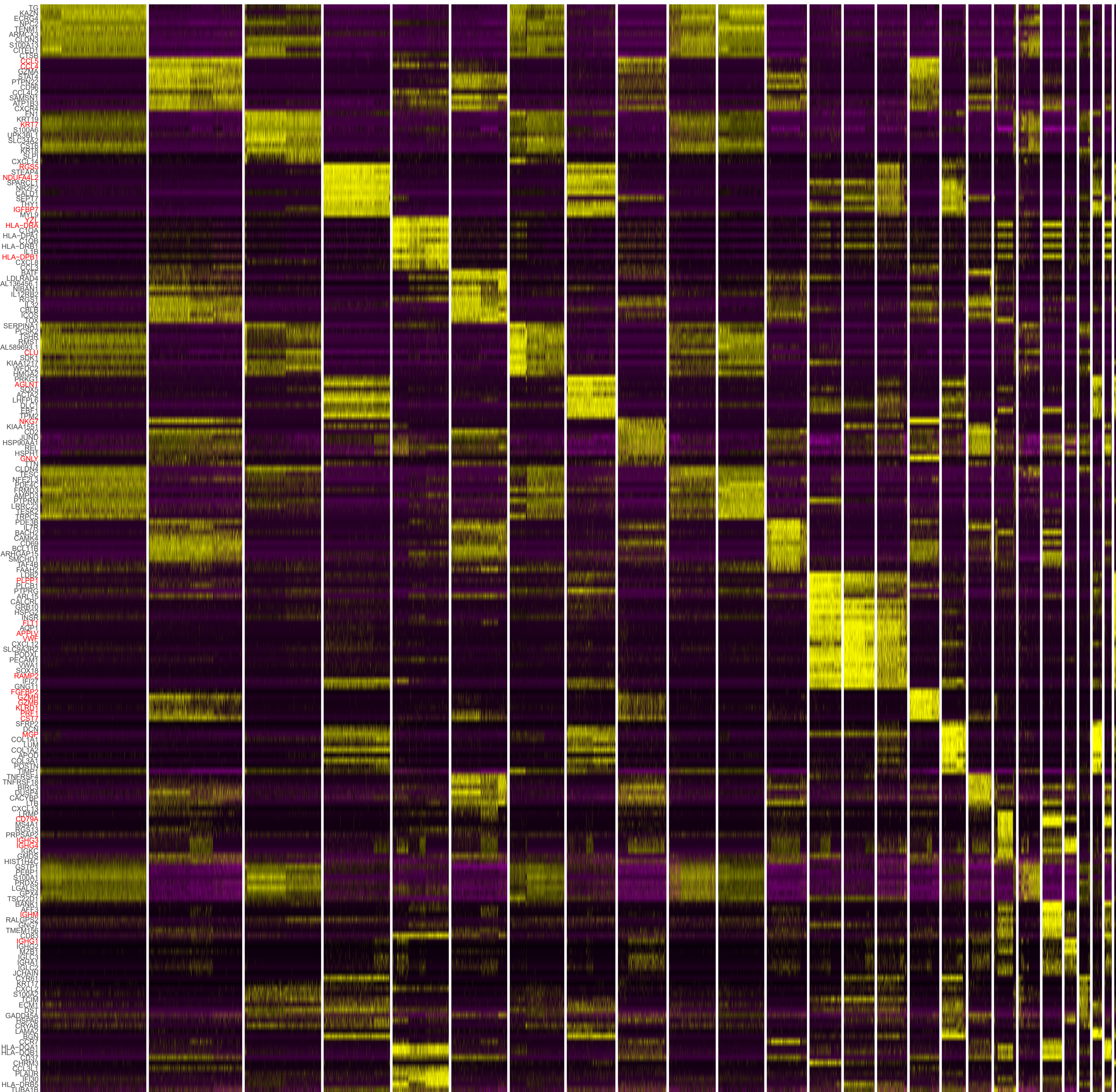

Supplement: Supplementary file 6 — Figure S4. The heatmap patterns for the top 100 DEGs across multiple cell types in the GSE191288 dataset. [file mmc4.pdf]

# Phylogenetic tree of clusters for GSE184362

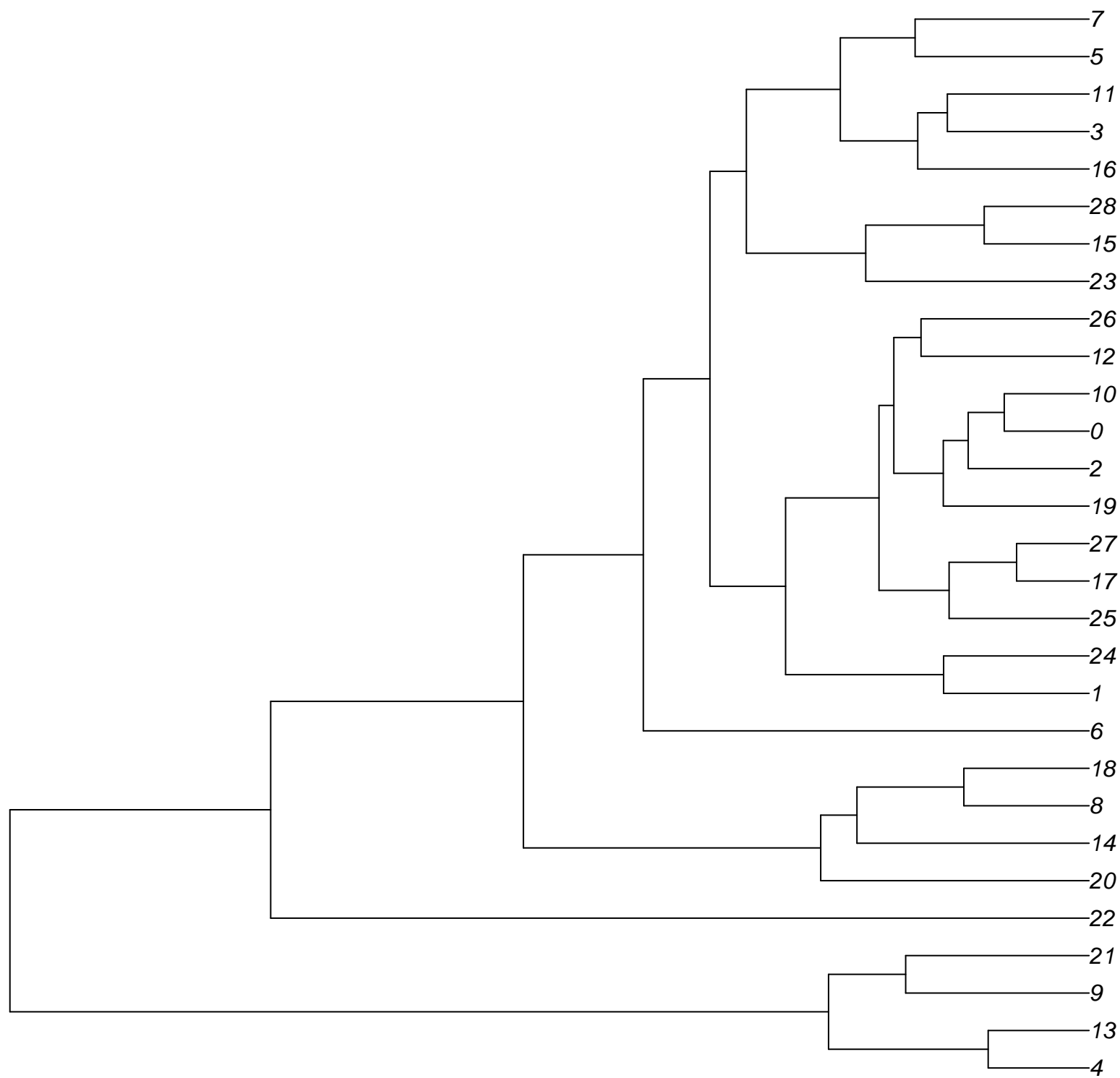

Supplement: Supplementary file 7 — Figure S5. The phylogenetic trees for the similarities between the cell clusters in GSE184362 dataset. [file mmc5.pdf]

# Phylogenetic tree of clusters for GSE191288

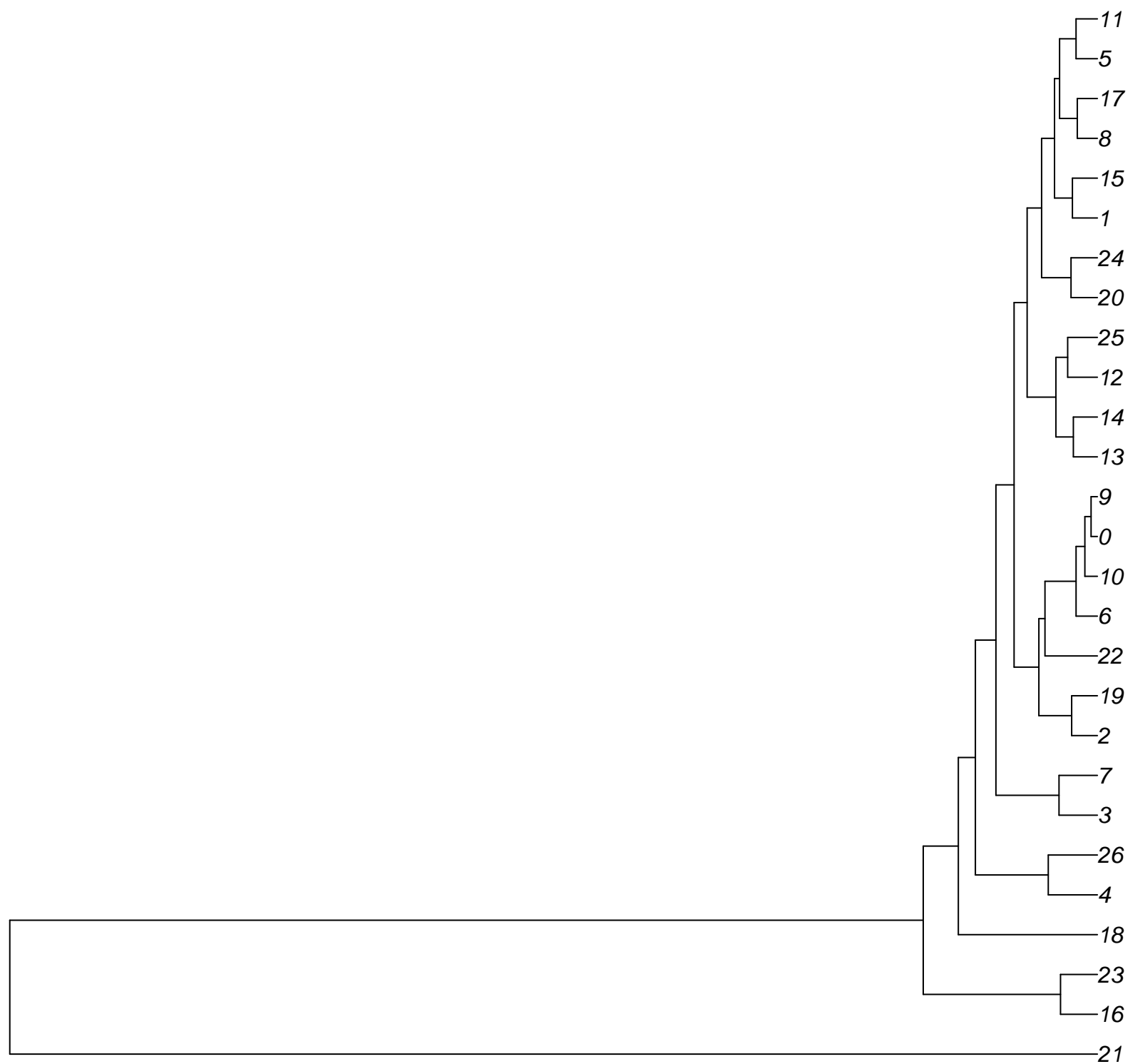

Supplement: Supplementary file 8 — Figure S6. The phylogenetic trees for the similarities between the cell clusters in GSE191288 dataset. [file mmc6.pdf]

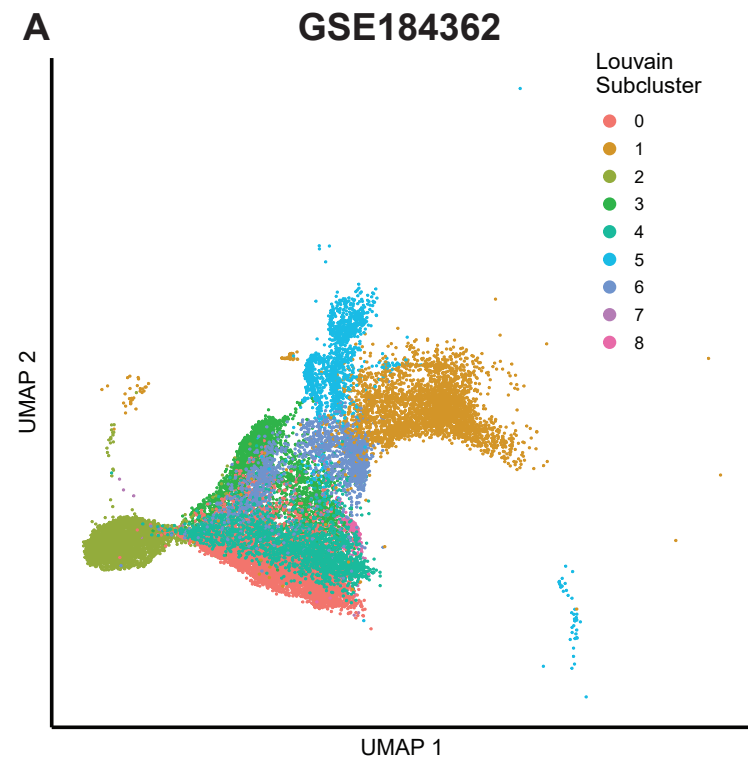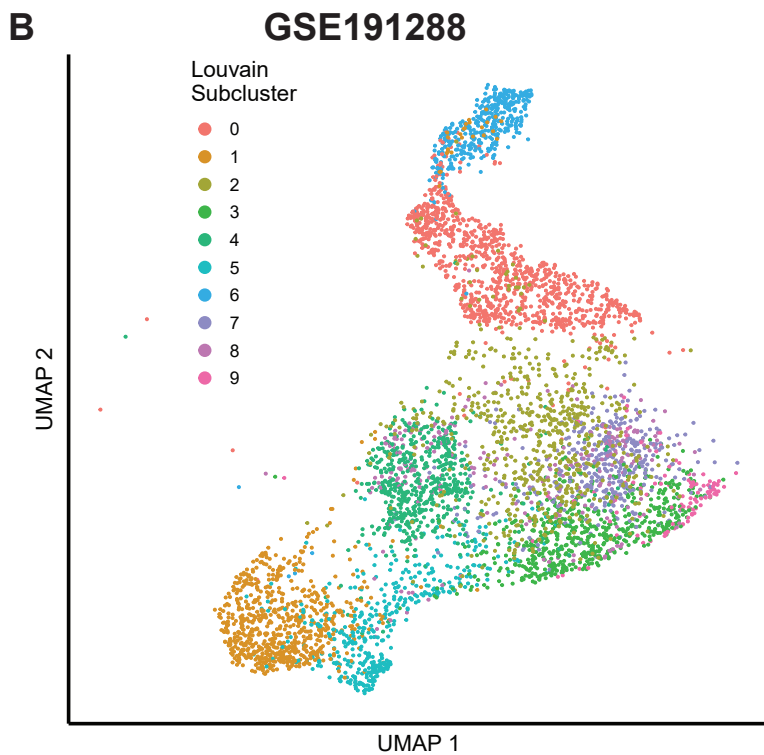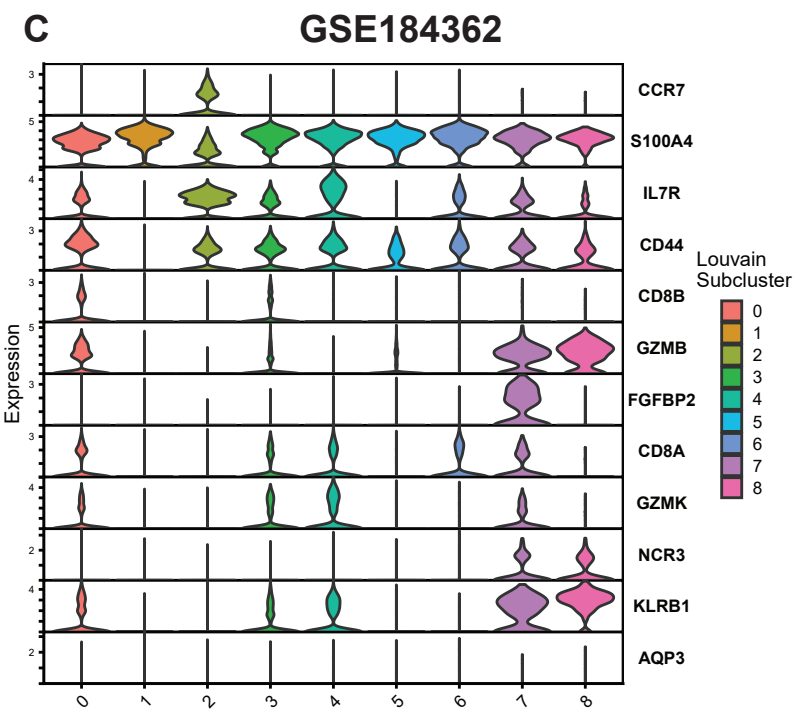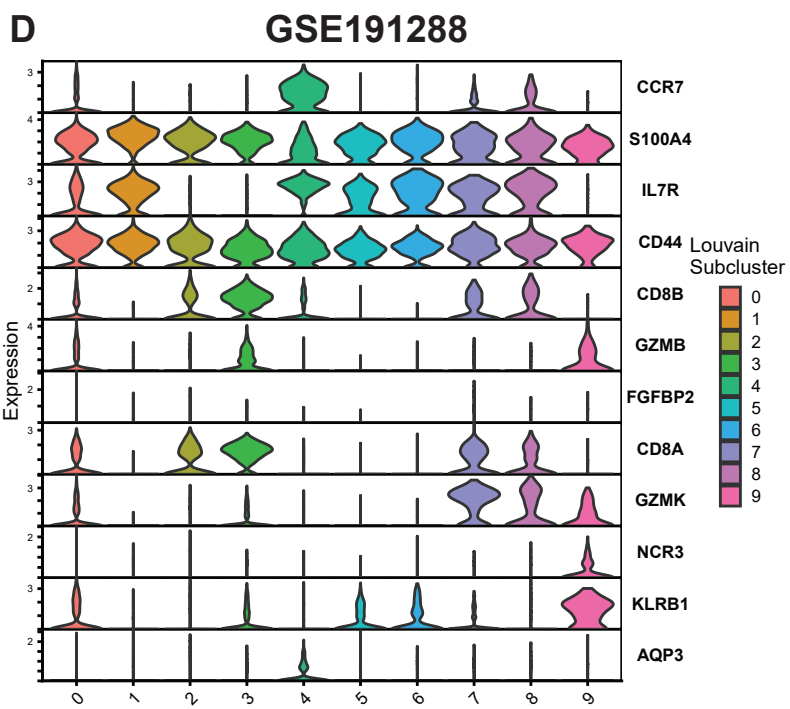

Supplement: Supplementary file 9 — Figure S7. Sub-clustering of T cells for two PTC scRNA-seq datasets. The UMAP visualization of T cells colored by Louvain sub-cluster for (A) GSE184362 and (B) GSE191288. The cluster ID were marked as numbers in the corresponding UMAP map. The violin plots depict the key marker genes for each cell cluster in (C) GSE184362 and (D) GSE191288. [file mmc7.pdf]
